# From Infection to Tumor: Exploring the Therapeutic Potential of Ciprofloxacin Derivatives as Anticancer Agents

**DOI:** 10.3390/ph18010072

**Published:** 2025-01-09

**Authors:** Hesham M. Hassan, Roket Hassan, Ranya Mohammed Elmagzoub, Ahmed Al-Emam, Konstantinos Kossenas, Ahmed S. Abdel-Samea, Hazim O. Khalifa, Suleyman Akocak, Stefan Bräse, Hamada Hashem

**Affiliations:** 1 Department of Pathology, College of Medicine, King Khalid University, Asir 61421, Saudi Arabia; 2 Department of Pathology, Faculty of Medicine, Assiut University, Assiut 71111, Egypt; 3 Department of Pharmaceutical Chemistry, Faculty of Pharmacy, Sohag University, Sohag 82524, Egypt; 4 Department of Medical Laboratory Technology, Faculty of Applied Medical Sciences, Northern Border University, Arar 73311, Saudi Arabia; 5 Department of Basic and Clinical Sciences, University of Nicosia Medical School, P.O. Box 24005, 21 Ilia Papakyriakou, 2414 Engomi, CY-1700 Nicosia, Cyprus; 6 Pharmacology & Toxicology Department, Faculty of Pharmacy, Deraya University, New Minia 61768, Egypt; 7 Department of Pharmacology, Faculty of Veterinary Medicinea, Kafrelsheikh University, Kafrelsheikh 33516, Egypt; 8 Department of Pharmaceutical Chemistry, Faculty of Pharmacy, Adıyaman University, Adıyaman 02040, Türkiye; 9 Institute of Biological and Chemical Systems-Functional Molecular Systems (IBCS-FMS), Karlsruhe Institute of Technology (KIT), Kaiserstrasse 12, 76131 Karlsruhe, Germany

**Keywords:** ciprofloxacin, anticancer, topoisomerases I and II inhibitors, apoptosis inducers, cell cycle arrest

## Abstract

Ciprofloxacin, a widely used second-generation fluoroquinolone for treating bacterial infections, has recently shown notable anticancer properties. This review explores progress in developing ciprofloxacin derivatives with anticancer properties, emphasizing key structural changes that improve their therapeutic effectiveness by modifying the basic group at position 7, the carboxylic acid group at position 3, or both. It further investigates the mechanisms by which these derivatives fight cancer, such as inducing apoptosis, arresting the cell cycle, inhibiting topoisomerase I and II, preventing tubulin polymerization, suppressing interleukin 6, blocking thymidine phosphorylase, inhibiting multidrug resistance proteins, and hindering angiogenesis. Additionally, it outlines their future directions, such as enhancing their efficacy, selectivity, and investigating potential synergy with other chemotherapeutic agents, offering a promising avenue for developing new therapies for cancer.

## 1. Introduction

Ciprofloxacin, a well-established fluoroquinolone antibiotic, plays a key role in treating numerous bacterial infections, including urinary tract infections, sexually transmitted diseases, typhoid fever, joint infections, gastrointestinal infections, prostatitis, lower respiratory tract infections, and salmonellosis, due to its potent activity against a broad spectrum of Gram-negative and Gram-positive bacteria [1,2,3,4]. Ciprofloxacin exerts its antibacterial effect mainly by inhibiting bacterial DNA gyrase and topoisomerase IV, which are crucial enzymes for DNA replication and transcription [5,6]. DNA topoisomerases are essential enzymes that control the topology of DNA, especially during key processes such as replication, transcription, and chromosome segregation [7,8,9].

**Figure 1:**
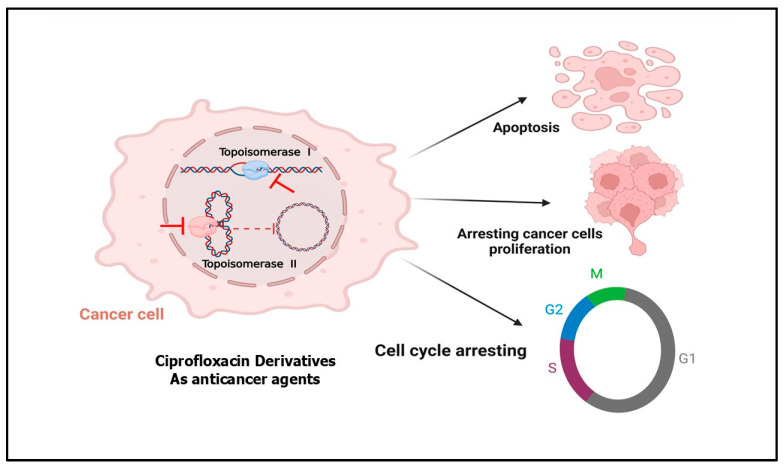
The schematic diagram depicts the anticancer mechanisms of ciprofloxacin derivatives, highlighting their role in inhibiting topoisomerases I and II within cancer cells, resulting in DNA damage, triggering apoptosis, and ultimately leading to the death of cells.

In vitro studies have demonstrated that ciprofloxacin can inhibit the proliferation of various cancer cell lines, including colon carcinoma lines (CC-531, SW-403, and HT-29) [10], transitional cell carcinoma lines (MBT-2 and T24), human lymphoid lines (Jurkat) [11], non-small-cell lung cancer lines (NCI-H460 and A549) [12], the hormone-resistant prostate cancer (HRPC) cell line (PC-3) [13], the ovarian cancer line (CHO AA8) [14], the bladder cancer line (HTB9), and the murine glioma line (GL26) [15]. Also, ciprofloxacin induces G2 phase arrest and apoptosis, highlighting its anticancer potential in various cancer cell lines [16,17,18]. In addition to its antiproliferative properties, ciprofloxacin’s well-established safety profile and pharmacokinetics have sparked significant interest among researchers for its potential repositioning from an antibacterial drug to an anticancer agent [19,20]. Furthermore, ciprofloxacin derivatives are regarded as promising anticancer agents targeting DNA topoisomerases [21,22]. Chemical modifications of ciprofloxacin could enhance its selectivity and potency in targeting topoisomerase I and II in cancer cells, offering a novel approach to disrupting cancer cell proliferation and survival [23]. As anticancer agents, ciprofloxacin derivatives may stabilize the cleavable complex between topoisomerase and DNA, thereby blocking the rejoining of DNA strands, resulting in DNA damage, triggering apoptosis, and ultimately leading to cancer cell death (Figure 1) [24,25,26].

This review provides a detailed overview of the recent advancements in developing ciprofloxacin derivatives with anticancer properties. It examines the potential mechanisms of anticancer activity for these derivatives. It discusses future directions to improve their efficacy and selectivity against cancer cells, aiming to inspire new anticancer therapies with reduced side effects.

## 2. Ciprofloxacin Physicochemical Properties

Ciprofloxacin, a second-generation fluoroquinolone antibiotic, possesses unique physicochemical properties contributing to its broad-spectrum antibacterial activity and efficacy in treating infections. It is a white, bitter-tasting compound with a melting point of 313–315 °C, requiring storage at 4 °C in the dark to prevent photodegradation. It has moderate water solubility influenced by pH, its solubility increases in acidic or basic environments due to its amphoteric nature. Ciprofloxacin is freely soluble in acetic acid but only slightly soluble in water, methanol, ethanol, or acetone. Its lipophilicity, measured by the partition coefficient (log P), aids in effective bacterial cell membrane penetration, while its stability over a range of pH levels supports its pharmacokinetic properties. The dissociation constants (pKa_1_ = 6.09 and pKa_2_ = 8.62) and an isoelectric point (pI = 7.14) reflect its two ionizable functional groups. The stronger acidity of the carboxylic group corresponds to pKa_1_, and the dissociation of a proton from the piperazine N-4 group corresponds to pKa_2_. At physiological pH, significant dissociation of both groups leads to the formation of zwitterionic species, enhancing its pharmacological effectiveness [27,28].

## 3. Essential Structure Modifications for Improving the Antiproliferative Activity of Ciprofloxacin

**Figure 2:**
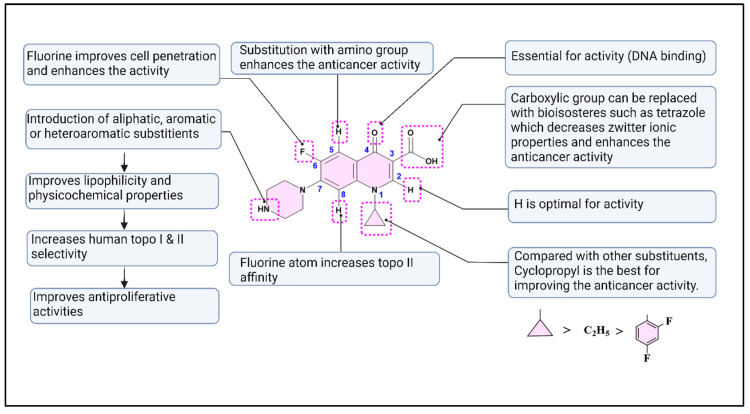
Illustrates the essential structural modifications of the ciprofloxacin core and their impact on anticancer activity and physicochemical properties, achieved either by introducing a substituent on the N-4 piperazine moiety, modifying the carboxylic acid group at position 3, or both.

Numerous studies have been conducted to identify the structural modifications required to shift ciprofloxacin activity from an antibacterial to an anticancer agent [29]. This can be achieved by reducing the zwitterionic nature of ciprofloxacin by altering the basic group at position 7, the carboxylic acid group at position 3, or both [26,30]. The nitrogen at position 1 of the quinolone ring is essential for its activity, and replacing the cyclopropyl group with an ethyl group has been shown to reduce anticancer potency [31]. The substituent at position 3 must remain in the same plane as the main quinolone structure, and having a hydrogen atom at position 2 is optimal for activity [21]. Similar to antibacterial activity, the carbonyl group at position 4 is essential for anticancer activity in some studies [3]. The introduction of an amino group at position 5 was reported to enhance the anticancer activity [3]. Fluorine atom at position 6 improves activity against mammalian topoisomerases [21]. The substituent on the N-4 position of the piperazine ring is essential, as it directly affects interactions with DNA topoisomerases, increases selectivity for mammalian enzymes, and markedly enhances anticancer activity [32]. The structure–activity relationship (SAR) for the anticancer activity of ciprofloxacin is summarized in (Figure 2).

## 4. Ciprofloxacin Derivatives with Anticancer Activity

In this context, enhancing the anticancer activity of ciprofloxacin has been extensively explored through structural modifications, specifically by the introduction of an aromatic or heteroaromatic substituent on its N-4 piperazine moiety, modifying the carboxylic acid group at C-3, or both [19,21].

### 4.1. N-4 Piperazine Modified Ciprofloxacin Derivatives

Altering the N-4-piperazine moiety of ciprofloxacin not only influences its physicochemical properties but also substantially enhances its anti-proliferative activity and improves the selectivity of ciprofloxacin derivatives for human topoisomerases [33]. As a result, numerous ciprofloxacin derivatives have been synthesized, characterized, and extensively studied for their antitumor potential. Mohammed and colleagues developed a series of N-4 piperazinyl ciprofloxacin derivatives as potential anticancer agents. Among these, compound **1** demonstrated notable activity against A549 non-small cell lung cancer cells with an IC_50_ of 14.8 μM, whereas the original ciprofloxacin showed only weak activity (IC_50_ > 100 μM). Although less potent than the reference drug doxorubicin (IC_50_ of 1 μM), ciprofloxacin derivative **1** selectively targeted cancer cells without harming normal WI-38 lung fibroblast cells. Its anticancer effect was linked to inducing G2/M cell cycle arrest by upregulating p53 and p21 proteins, thereby inhibiting cancer cell proliferation [18] Further studies on drug-resistant endometrial cancer cells revealed that compound **1** exerts its anticancer effects through multiple mechanisms, including the inhibition of topoisomerase I and II, which are essential for DNA replication and cell division, thus hindering cancer cell growth. It also enhanced the efficacy of the antimitotic drug paclitaxel (PTX) by promoting tubulin polymerization, leading to G2/M phase cell cycle arrest and apoptosis via caspase-3 activation. Additionally, ciprofloxacin derivative **1** inhibited the MDR1 efflux pump, which increases the intracellular concentration of PTX, aiding in overcoming drug resistance in cancer cells [24,34]. Similarly, Swedan and colleagues reported that ciprofloxacin derivative **2** demonstrated notable anticancer activity with IC_50_ values of 3.88 µM and 9.35 µM against T-24 bladder and PC-3 prostate cancer cell lines, respectively. Compared to the reference drug doxorubicin, ciprofloxacin derivative **2** was 1.2 to 7.1-fold more potent. Its anticancer mechanism involves inhibition of topoisomerase II, resulting in apoptosis through increased caspase-3 levels and cell cycle arrest [35]. Fallica and colleagues reported that compound **3** demonstrated significantly enhanced anticancer activity compared to its parent compound, ciprofloxacin, across multiple cancer cell lines. In DU145 prostate cancer cells, Compound **3** achieved an IC_50_ of 2.42 μM, substantially lower than ciprofloxacin’s 24.88 μΜ. A similar trend was observed in PC3 cells, where compound **3** exhibited an IC_50_ of 3.02 μM, far surpassing ciprofloxacin’s 33.90 μM. In MCF7 and MDA-MB231 breast cancer cells, compound **3** again outperformed ciprofloxacin, with IC_50_ values of 5.23 μM and 2.51 μM, respectively, compared to 8.85 μM and 19.88 μM for ciprofloxacin. The superior potency of compound **3** was attributed to its ability to inhibit topoisomerase II, thereby disrupting DNA replication and inducing apoptosis [36]. Al-Taweel et al. explored the anticancer properties of ciprofloxacin-1,2,3-triazole hybrids, Among the tested hybrids, ciprofloxacin derivative **4** exhibited remarkable anticancer effects, with IC_50_ values of 1.2 μM for U-87 glioblastoma, 10.58 μM for MCF-7 breast cancer, and 29.4 μM for A549 lung cancer cells. In comparison, the reference drug cisplatin showed higher IC_50_ values of 28.3 μM for U-87, 26.9 μM for MCF-7, and 30.4 μM for A549, making compound **4** more potent. Moreover, compound **4** demonstrated significantly lower toxicity toward normal HDF cells, with an IC_50_ of 170.7 μM, reflecting an improved therapeutic profile [23].

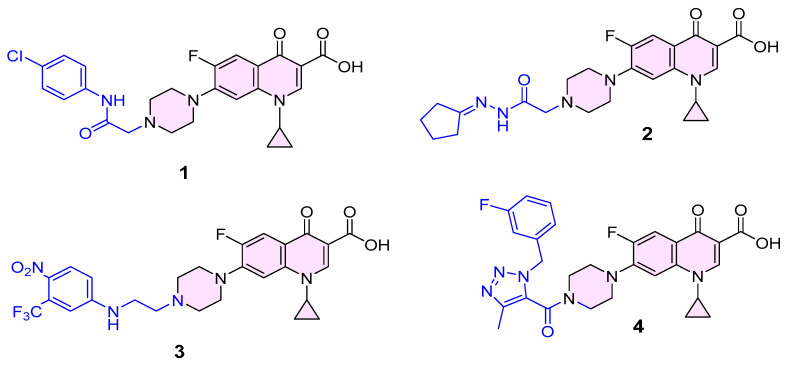



Shou and colleagues reported a series of new fluoroquinolone derivatives and evaluated their anticancer activities. Among them, ciprofloxacin derivative **5** revealed potent anticancer activity against HL-60 (leukemia), A549 (lung carcinoma), and HeLa (cervical cancer) cell lines with IC_50_ values of 0.04 µM, 0.07 µM, and 0.03 µM, respectively, which were comparable to the reference compound irinotecan (IC_50_: 0.03–0.04 µM across the same cell lines). The anticancer mechanism of compound **5** involved inhibition of DNA topoisomerase I, as confirmed by docking studies, which revealed strong binding interactions within the enzyme’s active site [37]. Mohammed and colleagues synthesized a series of urea-linked ciprofloxacin–chalcone hybrids, among which compound **6** displayed a remarkable antiproliferative effect with IC_50_ values of 2.01 µM for colon cancer HCT-116 cells and 0.64 µM for leukemia SR cells, outperforming camptothecin (IC_50_ = 17.36 µM and 3.32 µM, respectively). The anticancer mechanism of compound **6** involved dual inhibition of topoisomerase I (56.72% inhibition) and topoisomerase IIβ (60.06% inhibition), as well as induction of apoptosis via activating caspases-3, -8, and -9, and promoting cytochrome C release [38]. Abdel-Rahman and colleagues synthesized a series of Mannich bases of ciprofloxacin, with compound **7** demonstrating broad-spectrum anticancer activity. It exhibited GI_50_ values ranging from 2.5 to 6.79 µM across multiple cancer cell lines, showing notable selectivity for renal and prostate cancers with selectivity ratios of 0.17 and 6.64, respectively. The reference, doxorubicin, showed lower selectivity in these assays. The anticancer mechanism of compound **7** was attributed to the induction of apoptosis, supported by caspase-3 overexpression, leading to effective inhibition of cell proliferation [39]. Abdel-Aziz et al. reported a series of ciprofloxacin–chalcone hybrids as potential anticancer agents. Among these hybrids, compound **8** showed remarkable anticancer activity against various cancer cell lines with GI_50_ values ranging from 0.21 to 57.6 µM, demonstrating the highest selectivity towards leukemia with a selectivity ratio of 6.71. The anticancer mechanism of compound **8** involved the inhibition of both topoisomerase I and II enzymes, contributing to its potent antiproliferative effects [40]. Further studies on compound **8** showed remarkable anticancer activity against HepG2 (liver cancer) and MCF7 (breast cancer) cell lines with IC_50_ values for HepG2 cells were 22 µg/mL and 5.6 µg/mL after 24 and 48 h, respectively, while for MCF7 cells, the values were 54 µg/mL and 11.5 µg/mL. These values were lower compared to the reference drug doxorubicin, which showed IC_50_ values of 67.5 µg/mL for HepG2 and 269.5 µg/mL for MCF7 cells after 24 h. The anticancer mechanism of ciprofloxacin derivative **8** involved G2/M cell cycle arrest, pre-G1 apoptosis, upregulation of p53 and TNF-α, and downregulation of COX-2 [41].

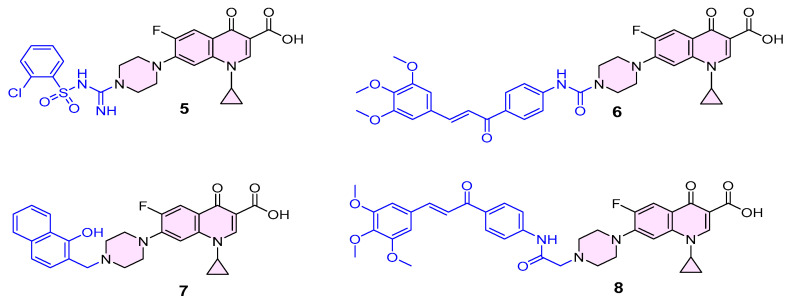



Demirci et al. evaluated the anticancer activity of a series of 1,3,4-thiadiazole-ciprofloxacin hybrids. At a concentration of 10 µM, compound **9** decreased cell viability to 81% in A549 lung cancer cells, while in MRC5 and HEK293 cell lines, viability was reduced to 63.2% and 66.8%, respectively. Compared to reference compounds like ciprofloxacin and norfloxacin, compound **9** demonstrated moderate anticancer activity. The proposed mechanism involved the inhibition of topoisomerase II, similar to other ciprofloxacin derivatives, which disrupts cell replication and survival [42,43]. Alaaeldin et al. investigated the anticancer activity of a novel ciprofloxacin derivative **10**, which exhibited cytotoxic effects against HepG2 and A549 cancer cells, with IC_50_ values of 22.09 µg/mL and 27.71 µg/mL, respectively. Etoposide was used as the reference compound, showing higher IC_50_ values of 34.32 µg/mL and 48.32 µg/mL for the same cell lines. The anticancer action of compound **10** was due to its dual inhibition of topoisomerase I and II, which caused DNA damage, induced cell cycle arrest at the G2/M phase, and triggered both apoptotic and necro-apoptotic pathways through the RIPK1/RIPK3/MLKL signaling cascade [25]. Mohammed et al. developed a series of ciprofloxacin–chalcone hybrids linked by 1,2,3-triazole. Among these, compound **11** displayed significant cytotoxic activity against colon cancer cell lines, with IC_50_ values of 2.53 µM in HCT116 cells, 13.24 µM in HT-29 cells, and 7.14 µM in Caco-2 cells. These values were compared to doxorubicin, which showed IC_50_ values of 1.22 µM, 0.88 µM, and 4.15 µM, respectively. Chalcone derivative **11** exhibited its anticancer effects by inhibiting topoisomerases I and II and disrupting tubulin polymerization. This action caused DNA damage and induced cell cycle arrest at the G2/M phase, eventually leading to apoptosis [22]. Fawzy et al. studied the anticancer activity of a novel ciprofloxacin derivative **12** on ovarian cancer (OVCAR-3) and lung cancer (A549) cell lines. Compound **12** exhibited IC_50_ values of 21.62 µM for OVCAR-3 and 32.98 µM for A549 cells, compared to the reference compound doxorubicin, which had IC_50_ values of 4.88 µM and 3.94 µM, respectively. Ciprofloxacin derivative **12** exhibits anticancer activity by inducing apoptosis and causing cell cycle arrest in the S-phase. It modulates the MAPK/ERK and p53/Bax/Bcl2 pathways, which together lead to decreased cell proliferation and enhanced programmed cell death [44].

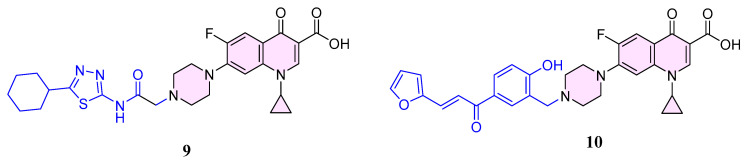

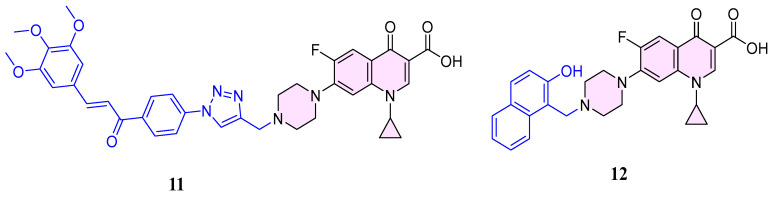





Struga and colleagues synthesized a series of N-4 substituted ciprofloxacin derivatives with promising antitumor potential. Among them, compound **13** demonstrated strong anticancer activity against PC3 prostate cancer cells, with an IC_50_ of 2.02 μM, making it 6.5 times more effective than cisplatin (IC_50_ of 13.1 μM). Additionally, the compound demonstrated reduced cytotoxicity toward normal HaCaT cells, with an IC_50_ of 35.07 μM. Its anticancer mechanism included triggering apoptosis and necrosis by elevating intracellular reactive oxygen species (ROS) levels and decreasing IL-6 in tumor cells [45]. Azéma and colleagues developed a series of ciprofloxacin derivatives with potential anticancer properties. Among them, compound **14** demonstrated notable anticancer effects, with IC_50_ values between 4 µM and 30 µM across various human cancer cell lines, including prostate (PC-3), non-small-cell lung cancer (A549), glioblastoma (U373-MG), colorectal (LoVo), and breast (MCF-7) cells. In contrast, the IC_50_ values of ciprofloxacin were much higher, generally exceeding 100 µM in these tests. The anticancer mechanism of compound **14** was linked to DNA topoisomerase II inhibition, resulting in apoptotic cell death [46]. Szostek and colleagues investigated the anticancer activity of menthol and thymol derivatives of ciprofloxacin. Menthol derivative **15** displayed effective anticancer activity across various cancer cell lines with IC_50_ values of 36.8 μM for HepG2, 27.1 μM for HCT-116, 30.3 μM for SW480, and 38.6 μM for SW620. These IC_50_ values indicate moderate potency relative to the reference drug doxorubicin, which exhibited IC_50_ values between 0.26 and 0.75 μM across the same cell lines. Unlike compound **15**, doxorubicin showed significantly higher cytotoxicity toward normal cells (HaCaT) [47]. Dileep and colleagues presented a series of N-4 piperazinyl tetrazole-ciprofloxacin hybrids. Among these hybrids, ciprofloxacin derivative **16** demonstrated significant anticancer activity, achieving GI_50_ values of 0.08 µM against the SiHa cell line, 0.22 µM against the MDA-MB-231 cell line, and 0.07 µM against the PANC-1 cell line. In comparison, the reference drug tamoxifen showed higher GI_50_ values of 0.12, 0.24, and 0.15 µM for the same cell lines, respectively [48].

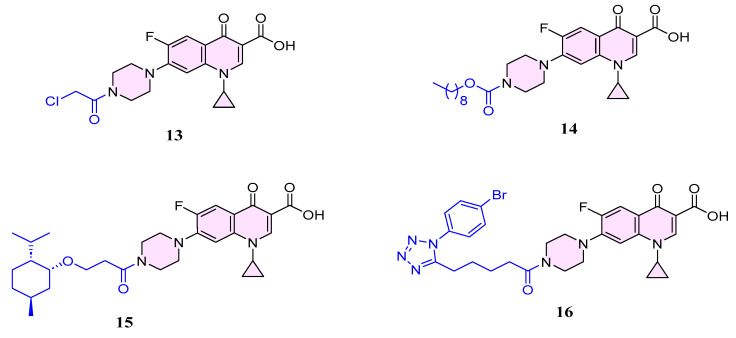



Farooqi and colleagues synthesized a series of aroylthiourea derivatives of ciprofloxacin as anticancer agents. Ciprofloxacin derivative **17** exhibited an IC_50_ value of 7.1 μM against the Huh-7 human hepatocellular carcinoma cell line. Compared to the reference drug ciprofloxacin, compound **17** showed more effective DNA binding and anticancer activity [49]. Piplani and colleagues synthesized a series of N-Mannich-based prodrugs of ciprofloxacin as potential anticancer agents. Among these prodrugs, compound **18** exhibited notable anticancer activity with a GI_50_ value of 28.8 µM against the A-549 human lung cancer cell line. In contrast, the reference drug Adriamycin showed considerably higher activity, with a GI_50_ value of less than 10 µM. The anticancer effect of compound **18** was linked to its increased lipophilicity, which facilitated greater penetration through cellular membranes, resulting in more effective inhibition of cancer cell growth compared to the parent drug [50]. Shahzad and colleagues evaluated the anticancer activity of a series of N-4 substituted ciprofloxacin derivatives as thymidine phosphorylase inhibitors. Among these derivatives, compound **19** has an IC_50_ value of 39.71 μM against thymidine phosphorylase. In comparison, the reference drug, 7-deazaxanthine, exhibited an IC_50_ of 37.82 μM. The anticancer effect of the compound was attributed to its inhibition of thymidine phosphorylase, an enzyme linked to tumor growth and angiogenesis [51]. Suresh and colleagues reported that compound **20** exhibited significant anticancer activity, showing comparable potency to the reference drug doxorubicin at a concentration of 50 µM across three human cancer cell lines: CCRF-CEM, MDA-MB-468, and HCT-116. The IC_50_ values for ciprofloxacin derivative **20** were not provided directly but it was effective in inhibiting cell proliferation by 35–60%. In comparison, doxorubicin (used at 10 µM) demonstrated similar activity. The anticancer mechanism of compound **20** was attributed to inhibition of topoisomerase II [43,52]

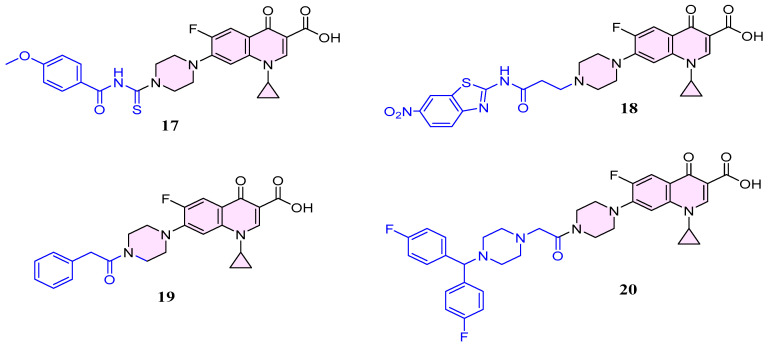



Ali et al. reported the anticancer potential of a new ciprofloxacin–chalcone hybrid **21** with IC_50_ values of 5.0 µM and 1.3 µM against HCT-116 and LOX IMVI cancer cells, respectively, outperforming the reference compound Staurosporine (IC_50_: 8.4 µM and 1.6 µM). The compound’s anticancer mechanism involved dual inhibition of topoisomerases I and II, cell cycle arrest at the G2/M phase, and apoptosis induction through increased expression of pro-apoptotic genes (Bax, Caspase 9) and decreased expression of the anti-apoptotic gene (Bcl-2) [53]. Foroumadi and colleagues evaluated a series of N-substituted piperazinyl ciprofloxacin derivatives as anticancer agents. Among these derivatives, compound **22** exhibited IC_50_ values of 7.7 µM against SKMEL-3 (melanoma), 2.9 µM against MCF-7 (breast carcinoma), 3.7 µM against A431 (epidermoid carcinoma), 3.9 µM against EJ (bladder carcinoma), 4.3 µM against SW480 (colon carcinoma), and 17 µM against KB (cervical carcinoma) cell lines. The reference drug etoposide had IC_50_ values ranging from 0.18 µM to 11.6 µM across the same cell lines. The anticancer mechanism of compound **22** was linked to the inhibition of DNA topoisomerase II, disrupting DNA replication and cell division [33]. Kassab et al. synthesized and evaluated a series of novel ciprofloxacin hybrids as potential cancer therapy. Among these hybrids, ciprofloxacin **23** exhibited strong anticancer activity, with IC_50_ values of 0.72 µM against the UO-31 renal cancer cell line, 0.75 µM against the IGROV1 ovarian cancer cell line, and 1.2 µM against the NCI-H226 non-small cell lung cancer line. These values show that compound **23** is 4.9 to 5.8 times more potent than the reference drug doxorubicin (IC_50_ values of 4.36 to 8.91 µM across these cell lines). The anticancer mechanism of compound **23** involved inhibition of topoisomerase II (IC_50_ of 0.86 µM) and induction of apoptosis via activation of caspase-3 [54]. In a study by Aziz et al., a series of thiazolidine-2,4-dione derivatives of ciprofloxacin were synthesized and evaluated as antiproliferative agents. Among the tested ciprofloxacin derivatives, compound **24** exhibited significant anticancer activity against the human melanoma LOX IMVI cancer cell line with an IC_50_ value of 25.4 µM, compared to the reference drugs doxorubicin and cisplatin, which had IC_50_ values of 7.03 and 5.07 µM, respectively. Mechanistically, ciprofloxacin derivative **24** inhibited both topoisomerase I and II enzymes with IC_50_ values of 4.77 µM and 15.00 µM, respectively. It also induced S-phase cell cycle arrest and triggered apoptosis, as evidenced by increased caspase-3 levels, enhanced Bax protein expression, and a reduction in PARP-1 activity [55].

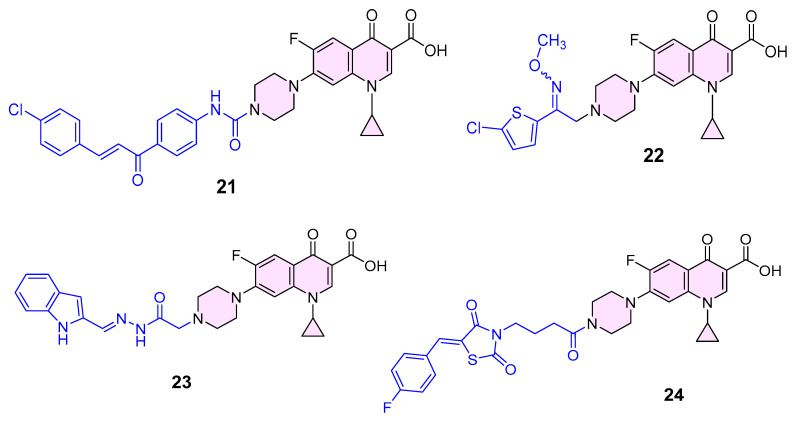



### 4.2. C-3-Modified Ciprofloxacin Derivatives

It has been observed that altering the carboxylic group of ciprofloxacin substantially improves its antiproliferative properties [56]. Regarding modifications at the C-3 position, the carboxylic acid group is crucial for the antibacterial activity of ciprofloxacin, primarily due to its role in DNA gyrase binding [9]. However, specific studies directly linking C-3 carboxylic acid modifications to changes in ROS levels are limited. The generation of ROS by fluoroquinolones, including ciprofloxacin, has been documented as a contributing factor to their antibacterial activity, and phototoxic effects. Also, these ROS can attack lipid membranes and cause DNA damage that results in tumor development. In this context, certain structural modifications have been explored to mitigate ROS-related toxicity. For example, incorporating phenyl thiourea moieties, known to react with ROS, has been investigated to alleviate phototoxic effects [57]. Ahadi et al. synthesized a series of ciprofloxacin-derived 1,3,4-thiadiazoles as anticancer agents. Among the tested compounds, thiadiazole derivative **25** demonstrated strong anticancer activity with IC_50_ values of 3.26 µM against MCF-7, 10.53 µM against A549, and 5.08 µM against SKOV-3 cancer cells, which is comparable to the reference drug doxorubicin, whose IC_50_ ranged from 2.25 to 3.48 µM across the same cell lines. The anticancer mechanism of compound **25** was primarily attributed to its ability to induce apoptosis and cause cell cycle arrest in the sub-G1 phase [58]. Shi et al. reported that the piperonal ciprofloxacin hydrazone **26** exhibited remarkable anticancer activity with IC_50_ values of 2.96 μM for SMMC-7721 hepatocarcinoma cells, 3.71 μM for MCF-7 breast cancer cells, and 3.69 μM for HCT-8 colon cancer cells. These values demonstrate significantly higher potency than ciprofloxacin, which had an IC_50_ of 6.86 μM in SMMC-7721 cells. The anticancer mechanism of Ciprofloxacin derivative **26** included the inhibition of topoisomerase II along with the induction of apoptosis [59].

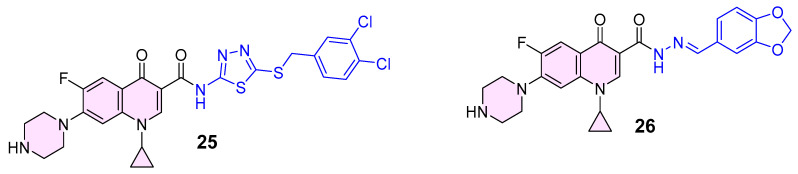



### 4.3. N-4 Piperazine- and C-3-Modified Ciprofloxacin Derivatives

Samir et al. synthesized a series of 3,7-bis-benzylidenes of ciprofloxacin as potential topoisomerases I and II inhibitors. Among these compounds, ciprofloxacin derivative **27** demonstrated significant anticancer activity with IC_50_ values of 1.21 µM for the leukemia HL-60 (TB) cell line, 0.87 µM for the colon cancer HCT-116 cell line, and 1.21 µM for the breast cancer MCF7 cell line. These IC_50_ values were comparable to the reference drug doxorubicin, which had IC_50_ values of 1.26 µM, 1.79 µM, and 0.63 µM, respectively, against the same cancer cell lines. The anticancer mechanism of compound **27** involved dual inhibition of topoisomerases Iα and IIB, with a % inhibition of 53.78% and 66.94%, respectively [60]. Hydroxamic acid, used to mimic the C3 carboxylic acid group, was introduced into ciprofloxacin. The resulting compounds were tested against four human cancer cell lines and demonstrated activity comparable to the SAHA reference drug. Among them, compounds **28** and **29** exhibited the strongest anticancer effects, with compound **28** being the most effective against pancreatic carcinoma PANC1 and lung adenocarcinoma A549. In contrast, compound **29** showed the highest potency against colon carcinoma HCT-116 and cervical adenocarcinoma HeLa [21]. Several studies have been conducted on ciprofloxacin derivatives designed as C3 bis-oxadiazole methyl sulfides. These derivatives were evaluated for their antitumor activity against human leukemia HL60, murine leukemia L1210, and Chinese hamster ovary (CHO). This modification significantly enhanced the anticancer activity, shifting them towards strong anticancer activity, resulting in compounds that were several times more potent than the original quinolones. Among the most active was compound **30**, which demonstrated GI_50_ values ranging from 1.1 to 2.8 µM. Likewise, C3-modified ciprofloxacin analogs, including compound **31**, exhibited notable antitumor potential when tested against hepatoma Hep-3B, leukemia HL60, and pancreatic ductal adenocarcinoma Capan-1 cell lines. Compound **31** demonstrated significantly greater activity than the anticancer drug doxorubicin, with IC_50_ values ranging from 0.3 to 1.5 µM, compared to doxorubicin’s range of 1.7 to 3.5 µM [21].

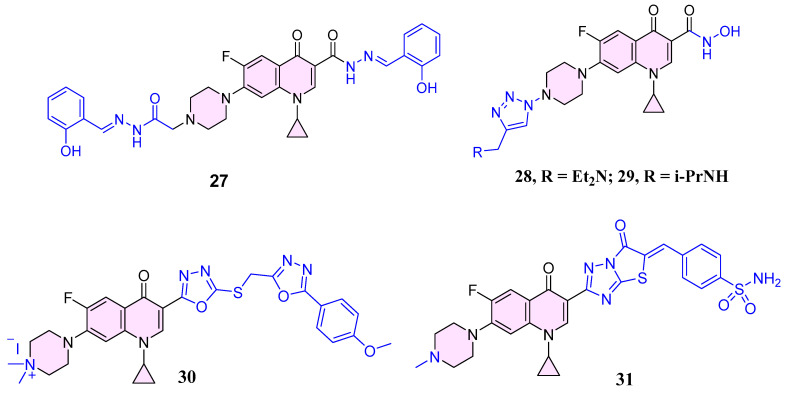



### 4.4. Ciprofloxacin Dimers

Azéma et al. reported the synthesis of Dimers of C7/C7 linked ciprofloxacin and evaluated them for their antitumor activity compared to their parent ciprofloxacin. Compound **32** demonstrated potent anticancer activity across several cancer cell lines with IC_50_ values ranging from 0.1 to 9 µM, significantly lower than the reference ciprofloxacin, whose IC_50_ values ranged from 89 to 476 µM depending on the cell line. Compound **32** showed enhanced potency, being 32 to 890 times more effective than the parent drug. The anticancer mechanism involves inhibiting DNA topoisomerases, leading to DNA damage and apoptosis in cancer cells [21]. Similarly, Hu et al. reported the synthesis and anticancer activity of C3/C3 linked ciprofloxacin derivatives via S-triazolo[2,1-b][1,3,4]thiadiazole moiety were evaluated along with the parent triazole quinolone **33.** Compound **33** showed moderate anticancer activity, with an IC_50_ of approximately 50 µM against L1210 murine leukemia cells. At the same time, derivatives **34**, **35**, and **36** exhibited significantly higher potency, with IC_50_ values of 0.20, 1.2, and 2.5 µM, respectively, against the same cell line. These compounds demonstrated much stronger activity than ciprofloxacin, which had IC_50_ values greater than 150 µM. The anticancer mechanism is attributed to the inhibition of topoisomerase II, leading to DNA damage and apoptosis in cancer cells [61].

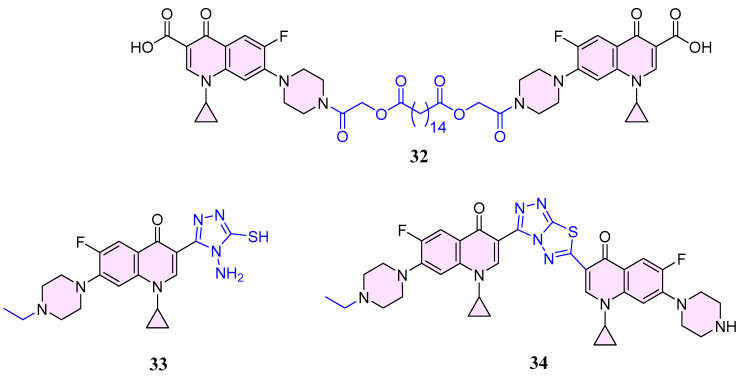

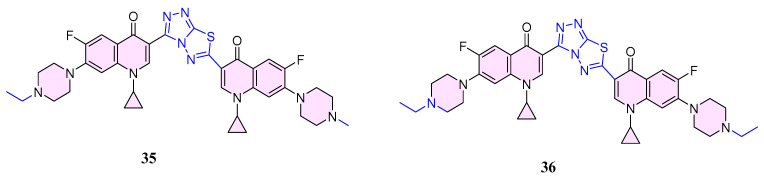





### 4.5. Ciprofloxacin Metal Complexes with Anticancer Activities

Metal ion complexes are of interest in medicinal chemistry due to their potential for inhibiting cell growth and anticancer activity [62,63]. Quinolones, specifically, act by forming ternary complexes with DNA and topoisomerases, facilitated by Mg^2+^ ions [64]. Additionally, metal ions are believed to contribute to topoisomerase poisoning by forming chelates with quinolones via the C3 and C4 oxygen atoms [65,66]. Incorporating ciprofloxacin into metal ion complexes has been shown to enhance their DNA binding, intercalation, and cytotoxic effects [67,68]. Studies have tested combinations of ciprofloxacin with 2,2′-bipyridyl derivatives, revealing strong cytotoxicity, DNA interaction, and superoxide radical scavenging. Among them, complexes **37** and **38** displayed the highest cytotoxic activity, with LC_50_ values of 6.2 µM and 6.0 µM, respectively [69]. Ciprofloxacin Cu(II) complexes with bidentate ligands displayed significant cytotoxicity, with compound **39** being the most potent. This complex demonstrated the ability to intercalate with DNA and exhibited free radical scavenging activity [70]. Copper (I) and (II) complexes with phosphine derivatives of fluoroquinolones, including ciprofloxacin complex **40**, were evaluated for their cytotoxic activity. Ciprofloxacin complex **40** was the most potent, with IC_50_ values ranging from 2.5 to 5.9 µM when tested on human A549 and murine colon carcinoma CT26 cell lines. This complex induced apoptosis, promoted reactive oxygen species formation, caused single-strand breaks, and intercalated with DNA [71].

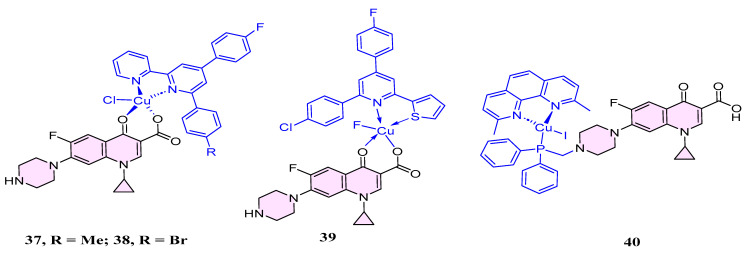



## 5. Potential Molecular Targets of Ciprofloxacin and Its Derivatives as Antiproliferative Agents

### 5.1. Inhibitors of Topoisomerases I/II Enzymes

**Figure 3:**
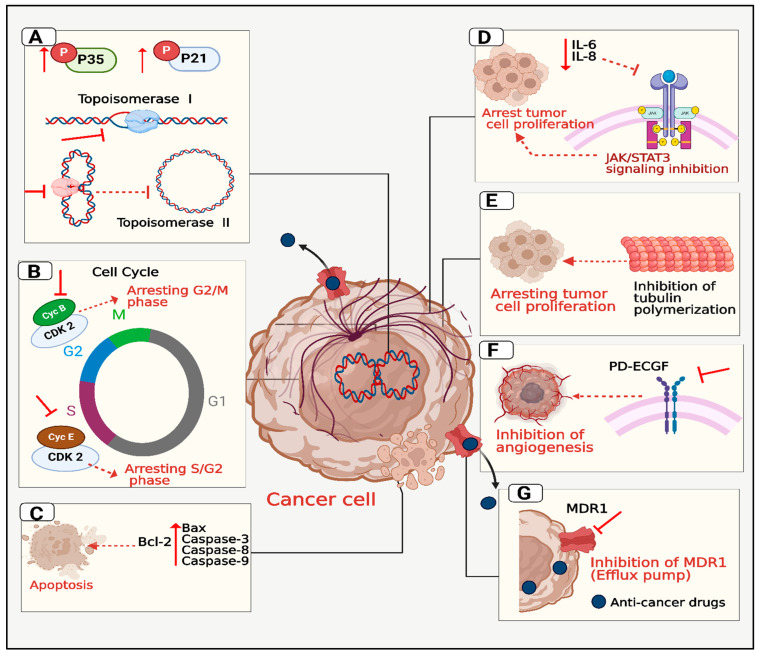
Illustration of potential molecular targets through which ciprofloxacin derivatives exert anticancer effects: (**A**) inhibiting topoisomerase I and II; (**B**) arresting the cell cycle; (**C**) inducing apoptosis; (**D**) blocking interleukin 6; (**E**) preventing tubulin polymerization; (**F**) impeding angiogenesis, and (**G**) inhibiting multidrug resistance proteins.

DNA topoisomerases are enzymes that manage DNA supercoiling during replication and transcription [72,73,74]. These enzymes function by creating single-strand breaks (type I) or double-strand breaks (type II) in DNA, followed by the rejoining of these breaks after allowing other DNA strands to pass through them [21]. In contrast, bacteria possess a unique topoisomerase called DNA gyrase, which introduces negative supercoils into DNA, a process crucial for DNA replication and compaction [75]. The structural and functional differences between human and bacterial topoisomerases have been exploited in medicine. Antibiotics like fluoroquinolones specifically target bacterial DNA gyrase, inhibiting bacterial replication without affecting human topoisomerases [76,77]. This selective inhibition is possible due to the distinct structural features of bacterial enzymes compared to their human counterparts [78]. Inhibiting prokaryotic DNA topoisomerases leads to antimicrobial effects while targeting their mammalian equivalents can serve as an effective approach for cancer treatment [21]. Due to structural and functional differences between human and bacterial topoisomerases, many studies investigated the possibility of repurposing ciprofloxacin from an antibacterial to an antitumor agent [79,80]. Recent studies highlight the role of fluoroquinolones as antiproliferative agents, with ciprofloxacin shown to inhibit topoisomerase II in mammalian cells [40,72]. SAR studies of ciprofloxacin indicate that modifying the N-4 position of its piperazine ring is crucial, as it directly influences interactions with DNA topoisomerases, enhances selectivity for mammalian enzymes, and significantly boosts anticancer activity [81,82,83]. Several ciprofloxacin derivatives with antiproliferative effects have been identified, targeting topoisomerase I/II enzymes (Figure 3A) [54,84].

### 5.2. Inducers of Cell Cycle Arrest

Cell growth and survival are controlled by cell cycle mechanisms, and recent studies have identified cancer as a disease of the cell cycle, marked by uncontrolled proliferation of tumor cells [85,86,87]. Ciprofloxacin has shown the ability to induce S/G2-phase arrest in prostate cancer cells and is recognized as the first fluoroquinolone to target the cyclin-CDK complex by reducing cyclins B/E and CDK2 levels in bladder cancer cells [88,89]. The tumor suppressor protein p53 is vital for arresting the cell cycle and induction of apoptosis in response to DNA damage, halting the cell cycle at the G2/M phase by activating target genes like p21 or directly inhibiting cyclins and CDKs [90,91]. Ciprofloxacin derivatives have also been found to induce p53/p21-mediated G2/M arrest in non-small cell lung cancer A549 cells (Figure 3B) [18].

### 5.3. Inducers of Apoptosis and Necro Apoptosis

Apoptosis, or programmed cell death, is a controlled process essential for eliminating cells, and its deficiency is often associated with cancer progression and chemotherapy resistance [92,93]. Key regulators of apoptosis, such as death receptors, Bcl-2 family proteins, caspases, and p53, are promising targets for chemotherapy [94]. Quite recently, ciprofloxacin and its derivatives were found to induce apoptosis by inhibiting topoisomerases I and II, causing DNA damage, increasing pro-apoptotic Bax, reducing anti-apoptotic Bcl2, and activating caspases [44,95]. Additionally, some ciprofloxacin derivatives can induce necro-apoptosis by activating the RIPK1/RIPK3/MLKL pathway (Figure 3C) [25].

### 5.4. Inhibitors of Interleukin-6 (IL-6) and IL-8

Interleukin-6 (IL-6) and interleukin-8 (IL-8) are pro-inflammatory cytokines crucial to cancer progression, promoting tumor growth, survival, and angiogenesis [96]. IL-6 activates the JAK/STAT3 pathway, supporting cell survival and resistance to apoptosis, while IL-8 aids tumor invasion, and metastasis [97,98,99,100]. Targeting these cytokines can inhibit tumor growth and improve chemotherapy outcomes [101,102]. Chrzanowska and colleagues developed ciprofloxacin-fatty acid conjugates that notably decreased IL-6 secretion in cancer cells, potentially inhibiting tumor progression and inflammation-associated cancer development (Figure 3D) [16].

### 5.5. Tubulin Polymerization Inhibitors

Tubulin polymerization, essential for cell division, forms the mitotic spindle that separates chromosomes during mitosis [103]. Cancer cells, due to their rapid division, depend heavily on this process, making it a key anticancer target [104]. Disrupting microtubule dynamics inhibits cancer cell proliferation by blocking spindle formation, leading to cell cycle arrest and apoptosis [105,106]. Mohammed et al. synthesized ciprofloxacin–chalcone hybrids that inhibit tubulin polymerization in HCT 116 colon cancer cells, inducing G2/M phase arrest and leading to apoptosis (Figure 3E) [22].

### 5.6. Thymidine Phosphorylase Inhibitors (Anti-Angiogenic Agents)

Thymidine phosphorylase (TP), also known as platelet-derived endothelial cell growth factor (PD-ECGF), plays a role in pyrimidine metabolism and has become a potential target in cancer therapy [107]. Commonly overexpressed in tumors, TP promotes cancer progression by stimulating angiogenesis, supplying tumors with essential oxygen and nutrients for growth and metastasis [108]. Additionally, TP contributes to chemotherapy resistance by altering the tumor microenvironment [109]. Inhibiting TP aims to reduce its pro-angiogenic effects, restrict tumor growth, and enhance anticancer treatments [110,111]. Shahzad and colleagues designed ciprofloxacin derivatives as anti-angiogenic agents, identifying nine compounds with notable TP inhibitory activity (Figure 3F) [51].

### 5.7. Inhibitor of Multidrug Resistance Protein 1 (MDR1)

The MDR1 efflux pump, or P-glycoprotein, often overexpressed in drug-resistant cancer cells, poses a significant challenge in cancer treatment by actively expelling chemotherapeutic drugs, reducing their intracellular concentrations and effectiveness [112]. This allows resistant cancer cells to survive, leading to poor therapeutic outcomes and recurrence [113]. Inhibiting MDR1 is a vital approach to overcoming drug resistance, as it allows therapeutic drugs to accumulate in tumor cells, enhancing their cytotoxicity [114]. A study by Alhag-Soluman and colleagues showed that a new ciprofloxacin derivative inhibited MDR1, increasing paclitaxel accumulation in drug-resistant endometrial cancer cells and boosting its efficacy (Figure 3G) [24].

### 5.8. Adjuvant Chemotherapy

Combination therapy, a strategy used in cancer treatment, enhances tumor cell death by targeting multiple pathways and reducing resistance risk [115]. Studies show that using ciprofloxacin with other anticancer drugs can boost treatment outcomes. For instance, pre-treatment with ciprofloxacin made resistant prostate cancer cells more susceptible to apoptosis when combined with etoposide [13]. Additionally, ciprofloxacin showed a synergistic effect with retinoic acid and Tamoxifen in hepatoma cells [10]. In vitro studies using a mouse model of human endometrial cancer demonstrated that a specific ciprofloxacin derivative, known as CIP2b, strengthened the effects of the antimitotic drug paclitaxel [24,34].

## 6. Future Directions of Ciprofloxacin Derivatives as Anticancer Agents

Ciprofloxacin, widely recognized as a broad-spectrum antibiotic, has recently gained attention for its potential applications as an anticancer agent. The rationale for exploring ciprofloxacin and its derivatives in oncology stems from their ability to inhibit topoisomerases I and II, induce DNA damage, and trigger apoptosis in a wide range of cancer cells. These mechanisms, vital for cancer cell proliferation and survival, offer promising avenues for future research and therapeutic development. Below are several potential directions and challenges in advancing ciprofloxacin derivatives for clinical applications.

### 6.1. Development of Targeted Derivatives

Ciprofloxacin, a fluoroquinolone antibiotic, can cause side effects such as nausea, diarrhea, tendon problems, nerve damage, central nervous system side effects, liver damage, skin sensitivity to ultraviolet light, heart rhythm problems, and seizures [31]. To reduce these side effects in anticancer applications, ongoing research aims to develop ciprofloxacin derivatives with greater selectivity and efficacy against cancer cells, involving chemical modifications to enhance binding affinity to cancer-specific targets. For instance, some ciprofloxacin derivatives can selectively inhibit mitochondrial DNA synthesis in cancer cells, minimizing off-target effects on healthy tissues. Further investigation into structure–activity relationships (SARs) will be crucial to optimize these compounds for targeting specific cancers.

### 6.2. Utilizing Nanotechnology-Based Drug Delivery Systems

Nanoparticle-based systems present a promising solution, as ciprofloxacin derivatives can be encapsulated in liposomes, polymeric nanoparticles, or other nanocarriers equipped with targeting ligands like antibodies or peptides. This strategy can increase bioavailability, reduce systemic toxicity, and improve tumor penetration. Further research on conjugating ciprofloxacin derivatives with nanotechnology platforms, such as dendrimers or micelles, could greatly enhance their therapeutic potential. Naguib and colleagues developed a combination therapy using CIP2b-loaded PEGylated polymeric nanoparticles (CIP2b-NPs) and PTX to treat type-II endometrial cancer with p53 loss. CIP2b-NPs synergistically enhanced PTX’s antitumor effects while demonstrating a favorable safety profile in vitro, with no toxicity observed. Intravenous delivery of CIP2b-NPs significantly improved tumor accumulation of CIP2b, boosting PTX’s efficacy at the tumor site. This approach slowed tumor progression and may reduce PTX-related side effects by enabling lower doses [34].

### 6.3. Synergistic Combinations with Other Chemotherapeutic Agents

Another promising strategy involves combining ciprofloxacin derivatives with existing chemotherapeutic agents to harness potential synergistic effects while reducing possible side effects. For instance, pairing ciprofloxacin with DNA-damaging agents like cisplatin or with inhibitors of specific cancer signaling pathways may boost efficacy while lowering the required doses of each drug. The ability of ciprofloxacin derivatives to modulate topoisomerase II, which is traditionally targeted by chemotherapeutic agents, offers an avenue to enhance the effectiveness of these treatments, especially in drug-resistant cancers. Alhaj-Suliman and colleagues investigated the combined use of the ciprofloxacin derivative CIP2b and paclitaxel (PTX) to combat resistance in type-II endometrial cancer associated with p53 mutations and MDR1 overexpression. In vitro studies demonstrated that the PTX + CIP2b combination significantly enhanced PTX accumulation in tumors, resulting in reduced tumor growth. Histological and immunohistochemical analyses confirmed the improved cytotoxic effects, while complete blood count and biochemistry tests showed no evidence of off-target toxicity. These results emphasize the potential of CIP2b and PTX as a safe and effective combination therapy for overcoming chemoresistance and enhancing cancer treatment outcomes [24].

### 6.4. Personalized Medicine

The use of ciprofloxacin derivatives in personalized medicine offers a promising step forward in oncology. These agents can be specifically designed to target molecular pathways in cancer cells, such as inhibiting topoisomerases or selectively inducing oxidative stress in tumor environments. By leveraging genetic or molecular biomarkers, clinicians can predict individual patient responses and tailor treatments to enhance effectiveness while minimizing side effects. Additionally, ciprofloxacin derivatives show potential in combination therapies, where their use alongside other drugs can improve patient-specific therapeutic outcomes. This targeted approach not only enhances treatment success but also aligns with efforts to reduce toxicity and improve cancer patients’ quality of life. With further research and development, these derivatives could become integral to advancing precision oncology.

### 6.5. Preclinical and Clinical Evaluation

Although in vitro studies and some in vitro models suggest promising anticancer potential for ciprofloxacin derivatives, a significant gap remains in translating these effects into clinical applications. Future research should prioritize thorough preclinical evaluations, encompassing toxicity, pharmacokinetics, and pharmacodynamics, to determine their feasibility for clinical trials. Furthermore, exploring the safety and efficacy of these derivatives in combination with other drugs is essential to optimize therapeutic strategies.

### 6.6. Challenges to Overcome

The development of ciprofloxacin derivatives as anticancer agents faces several challenges that must be overcome to unlock their full therapeutic potential. A primary concern is the risk of resistance, as prolonged exposure to these derivatives may lead to resistance mechanisms. To counter this, focused research is needed to understand, prevent, and counteract these resistance pathways, ensuring long-term efficacy. Another major challenge is the regulatory complexity involved in repurposing antibiotics for cancer treatment. Moving ciprofloxacin derivatives from experimental studies to clinical use demands rigorous testing to confirm both safety and effectiveness in oncology, a process that is both intricate and resource intensive. Furthermore, these derivatives must undergo extensive clinical trials to assess pharmacokinetics, optimize dosing, and minimize adverse effects for cancer patients. Overcoming these challenges will require dedicated preclinical and clinical research, as well as close collaboration with regulatory bodies to facilitate a smooth approval process.

## 7. Conclusions

In conclusion, transforming ciprofloxacin from an antibacterial agent into an antitumor compound through chemical modification offers an exciting new direction in the development of anticancer drugs. SAR studies have identified essential structural changes that boost the anticancer efficacy of ciprofloxacin, such as altering the basic group at position 7, the carboxylic acid group at position 3, or both. Structural modifications of ciprofloxacin have led to the development of compounds with increased cytotoxic activity against various cancer cell lines, demonstrating a promising ability to selectively target cancer cells while maintaining minimal toxicity to normal cells. Mechanistically, these derivatives combat cancer through various mechanisms such as inducing apoptosis, arresting the cell cycle, inhibiting topoisomerase I and II, preventing tubulin polymerization, suppressing interleukin 6, blocking thymidine phosphorylase, inhibiting multidrug resistance proteins, and hindering angiogenesis. Although preclinical studies show the potential of ciprofloxacin derivatives as anticancer agents, additional in vitro studies and pharmacokinetic assessments are needed to validate their ADME profiles relative to that of the parent ciprofloxacin. Also, clinical trials are essential to fully evaluate the safety, efficacy, and therapeutic potential of these derivatives. Moreover, challenges such as drug resistance mechanisms and possible off-target effects warrant further investigation. As research progresses, ciprofloxacin derivatives may emerge as valuable additions to the arsenal of anticancer agents, bringing renewed hope in tackling resistant and aggressive cancers.

## Data Availability

The data can be shared upon request.

## References

[1] Al-Hakkani M.F., Ahmed N., Abbas A.A., Hassan M.H.A., Aziz H.A., Elshamsy A.M., Khalifa H.O., Abdelshakour M.A., Saddik M.S., Elsayed M.M.A. (2023). Synthesis, Physicochemical Characterization using a Facile Validated HPLC Quantitation Analysis Method of 4-Chloro-phenylcarbamoyl-methyl Ciprofloxacin and Its Biological Investigations. Int. J. Mol. Sci..

[2] Mohammed H.H.H., Abbas S.H., Abdelhafez E.-S.M.N., Berger J.M., Mitarai S., Arai M., Abuo-Rahma G.E.-D.A.A. (2019). Synthesis, molecular docking, antimicrobial evaluation, and DNA cleavage assay of new thiadiazole/oxadiazole ciprofloxacin derivatives. Monatshefte Chem..

[3] Mohammed H.H.H., Abuo-Rahma G.E.-D.A.A., Abbas S.H., Abdelhafez E.-S.M.N. (2019). Current Trends and Future Directions of Fluoroquinolones. Curr. Med. Chem..

[4] Thai T., Salisbury B.H., Zito P.M. (2024). Ciprofloxacin. StatPearls.

[5] Mohammed H.H.H., Abdelhafez E.-S.M.N., Abbas S.H., Moustafa G.A.I., Hauk G., Berger J.M., Mitarai S., Arai M., El-Baky R.M.A., Abuo-Rahma G.E.-D.A. (2019). Design, synthesis and molecular docking of new N-4-piperazinyl ciprofloxacin-triazole hybrids with potential antimicrobial activity. Bioorganic Chem..

[6] Aziz H.A., El-Saghier A.M.M., Badr M., Abuo-Rahma G.E.-D.A., Shoman M.E. (2021). Thiazolidine-2,4-dione-linked ciprofloxacin derivatives with broad-spectrum antibacterial, MRSA and topoisomerase inhibitory activities. Mol. Divers..

[7] McKinnon P.J. (2016). Topoisomerases and the regulation of neural function. Nat. Rev. Neurosci..

[8] McKie S.J., Neuman K.C., Maxwell A. (2021). DNA topoisomerases: Advances in understanding of cellular roles and multi-protein complexes via structure-function analysis. BioEssays.

[9] Aziz H.A., Moustafa G.A.I., Abuo-Rahma G.E.-D.A., Rabea S.M., Hauk G., Krishna V.S., Sriram D., Berger J.M., Abbas S.H. (2021). Synthesis and antimicrobial evaluation of new nitric oxide-donating fluoroquinolone/oxime hybrids. Arch. Der Pharm..

[10] Herold C., Ocker M., Ganslmayer M., Gerauer H., Hahn E.G., Schuppan D. (2002). Ciprofloxacin induces apoptosis and inhibits proliferation of human colorectal carcinoma cells. Br. J. Cancer.

[11] Koziel R., Szczepanowska J., Magalska A., Piwocka K., Duszynski J., Zablocki K. (2010). Ciprofloxacin inhibits proliferation and promotes generation of aneuploidy in Jurkat cells. J. Physiol. Pharmacol..

[12] Kloskowski T., Gurtowska N., Olkowska J., Nowak J.M., Adamowicz J., Tworkiewicz J., Dębski R., Grzanka A., Drewa T. (2012). Ciprofloxacin is a potential topoisomerase II inhibitor for the treatment of NSCLC. Int. J. Oncol..

[13] El-Rayes B.F., Grignon R., Aslam N., Aranha O., Sarkar F.H. (2002). Ciprofloxacin inhibits cell growth and synergises the effect of etoposide in hormone resistant prostate cancer cells. Int. J. Oncol..

[14] Kloskowski T., Olkowska J., Nazlica A., Drewa T. (2010). The influence of ciprofloxacin on hamster ovarian cancer cell line CHO AA8. Acta Pol. Pharm..

[15] Esmaeilzadeh A. (2012). Influence of ciprofloxacin on glioma cell line GL26: A new application for an old antibiotic. Afr. J. Microbiol. Res..

[16] Chrzanowska A., Roszkowski P., Bielenica A., Olejarz W., Stępień K., Struga M. (2019). Anticancer and antimicrobial effects of novel ciprofloxacin fatty acids conjugates. Eur. J. Med. Chem..

[17] Gao Y., Shang Q., Li W., Guo W., Stojadinovic A., Mannion C., Man Y., Chen T. (2020). Antibiotics for cancer treatment: A double-edged sword. J. Cancer.

[18] Mohammed H.H.H., El-Hafeez A.A.A., Abbas S.H., Abdelhafez E.-S.M.N., Abuo-Rahma G.E.-D.A. (2016). New antiproliferative 7-(4-(N-substituted carbamoylmethyl)piperazin-1-yl) derivatives of ciprofloxacin induce cell cycle arrest at G2/M phase. Bioorganic Med. Chem..

[19] Yadav V., Talwar P. (2019). Repositioning of fluoroquinolones from antibiotic to anti-cancer agents: An underestimated truth. Biomed. Pharmacother..

[20] Mo X., Rao D.P., Kaur K., Hassan R., Abdel-Samea A.S., Farhan S.M., Bräse S., Hashem H. (2024). Indole Derivatives: A Versatile Scaffold in Modern Drug Discovery—An Updated Review on Their Multifaceted Therapeutic Applications (2020–2024). Molecules.

[21] Abdel-Aal M.A.A., Abdel-Aziz S.A., Shaykoon M.S.A., Abuo-Rahma G.E.-D.A. (2019). Towards anticancer fluoroquinolones: A review article. Arch. Pharm..

[22] Mohammed H.H.H., El-Hafeez A.A.A., Ebeid K., Mekkawy A.I., Abourehab M.A.S., Wafa E.I., Alhaj-Suliman S.O., Salem A.K., Ghosh P., Abuo-Rahma G.E.-D.A. (2022). New,3-triazole linked ciprofloxacin-chalcones induce DNA damage by inhibiting human topoisomerase I& II and tubulin polymerization. J. Enzym. Inhib. Med. Chem..

[23] Al-Taweel S., Al-Saraireh Y., Al-Trawneh S., Alshahateet S., Tarawneh R.A., Ayed N., Alkhojah M., AL-Khaboori W., Zereini W., Al-Qaralleh O. (2023). Synthesis and biological evaluation of ciprofloxacin—1,2,3-triazole hybrids as antitumor, antibacterial, and antioxidant agents. Heliyon.

[24] Alhaj-Suliman S.O., Naguib Y.W., Wafa E.I., Saha S., Ebeid K., Meng X., Mohammed H.H., Abuo-Rahma G.E.-D.A., Yang S., Salem A.K. (2023). A ciprofloxacin derivative with four mechanisms of action overcomes paclitaxel resistance in p53-mutant and MDR1 gene-expressing type II human endometrial cancer. Biomaterials.

[25] Alaaeldin R., Abdel-Rahman I.M., Ali F.E.M., Bekhit A.A., Elhamadany E.Y., Zhao Q.-L., Cui Z.-G., Fathy M. (2022). Dual Topoisomerase I/II Inhibition-Induced Apoptosis and Necro-Apoptosis in Cancer Cells by a Novel Ciprofloxacin Derivative via RIPK1/RIPK3/MLKL Activation. Molecules.

[26] Sharma P.C., Goyal R., Sharma A., Sharma D., Saini N., Rajak H., Sharma S., Thakur V.K. (2020). Insights on fluoroquinolones in cancer therapy: Chemistry and recent developments. Mater. Today Chem..

[27] Sharma P.C., Jain A., Jain S., Pahwa R., Yar M.S. (2010). Ciprofloxacin: Review on developments in synthetic, analytical, and medicinal aspects. J. Enzym. Inhib. Med. Chem..

[28] Masoumi B., Eslami G., Alizadeh-Navaei R., Mondal P., Rezai M.S. (2019). Safety Profile of Using Ciprofloxacin in Paediatrics: A Systematic Review and Meta-Analysis. J. Pediatr. Rev..

[29] Sissi C., Palumbo M. (2003). The quinolone family: From antibacterial to anticancer agents. Curr. Med. Chem. Agents.

[30] Suaifan G.A.R.Y., Mohammed A.A.M. (2019). Fluoroquinolones structural and medicinal developments (2013–2018): Where are we now?. Bioorganic Med. Chem..

[31] Nowakowska J., Radomska D., Czarnomysy R., Marciniec K. (2024). Recent Development of Fluoroquinolone Derivatives as Anticancer Agents. Molecules.

[32] Alovero F.L., Pan X.-S., Morris J.E., Manzo R.H., Fisher L.M. (2000). Engineering the Specificity of Antibacterial Fluoroquinolones: Benzenesulfonamide Modifications at C-7 of Ciprofloxacin Change Its Primary Target in Streptococcus pneumoniae from Topoisomerase IV to Gyrase. Antimicrob. Agents Chemother..

[33] Foroumadi A., Emami S., Rajabalian S., Badinloo M., Mohammadhosseini N., Shafiee A. (2009). N-Substituted piperazinyl quinolones as potential cytotoxic agents: Structure–activity relationships study. Biomed. Pharmacother..

[34] Naguib Y.W., Alhaj-Suliman S.O., Wafa E.I., Saha S., Ebeid K., Mohammed H.H.H., Abdel-Rahman S.A., Abuo-Rahma G.E.-D.A., Geary S.M., Salem A.K. (2024). Ciprofloxacin Derivative-Loaded Nanoparticles Synergize with Paclitaxel Against Type II Human Endometrial Cancer. Small.

[35] Swedan H.K., Kassab A.E., Gedawy E.M., Elmeligie S.E. (2023). Design, synthesis, and biological evaluation of novel ciprofloxacin derivatives as potential anticancer agents targeting topoisomerase II enzyme. J. Enzym. Inhib. Med. Chem..

[36] Fallica A.N., Barbaraci C., Amata E., Pasquinucci L., Turnaturi R., Dichiara M., Intagliata S., Gariboldi M.B., Marras E., Orlandi V.T. (2021). Nitric Oxide Photo-Donor Hybrids of Ciprofloxacin and Norfloxacin: A Shift in Activity from Antimicrobial to Anticancer Agents. J. Med. Chem..

[37] Shou K.-J., Li J., Lv Y.-W. (2013). Design, synthesis, biological evaluation, and molecular docking studies of quinolone derivatives as potential antitumor topoisomerase I inhibitors. Chem. Pharm. Bull..

[38] Mohammed H.H.H., Abbas S.H., Hayallah A.M., Abuo-Rahma G.E.-D.A., Mostafa Y.A. (2021). Novel urea linked ciprofloxacin-chalcone hybrids having antiproliferative topoisomerases I/II inhibitory activities and caspases-mediated apoptosis. Bioorganic Chem..

[39] Abdel-Rahman I.M., Mustafa M., Mohamed S.A., Yahia R., Abdel-Aziz M., Abuo-Rahma G.E.-D.A., Hayallah A.M. (2021). Novel Mannich bases of ciprofloxacin with improved physicochemical properties, antibacterial, anticancer activities and caspase-3 mediated apoptosis. Bioorganic Chem..

[40] Abdel-Aziz M., Park S.-E., Abuo-Rahma G.E.-D.A.A., Sayed M.A., Kwon Y. (2013). Novel N-4-piperazinyl-ciprofloxacin-chalcone hybrids: Synthesis, physicochemical properties, anticancer and topoisomerase I and II inhibitory activity. Eur. J. Med. Chem..

[41] Eisa M.A., Fathy M., Abuo-Rahma G.E.-D.A.A., Abdel-Aziz M., Nazmy M.H. (2021). Anti-Proliferative and Pro-Apoptotic Activities of Synthesized 3,4,5 Tri-Methoxy Ciprofloxacin Chalcone Hybrid, through p53 Up-Regulation in HepG2 and MCF7 Cell Lines. Asian Pac. J. Cancer Prev..

[42] Demirci A., Karayel K., Tatar E., Okullu S., Ünübol N., Taşli P., Kocagöz Z., Şahin F., Küçükgüzel İ. (2018). Synthesis and evaluation of novel 1,3,4-thiadiazole--fluoroquinolone hybrids as antibacterial, antituberculosis, and anticancer agents. Turk. J. Chem..

[43] Swedan H.K., Kassab A.E., Gedawy E.M., Elmeligie S.E. (2023). Topoisomerase II inhibitors design: Early studies and new perspectives. Bioorganic Chem..

[44] Fawzy M.A., Abu-Baih R.H., Abuo-Rahma G.E.-D.A., Abdel-Rahman I.M., El-Sheikh A.A.K., Nazmy M.H. (2023). In Vitro Anticancer Activity of Novel Ciprofloxacin Mannich Base in Lung Adenocarcinoma and High-Grade Serous Ovarian Cancer Cell Lines via Attenuating MAPK Signaling Pathway. Molecules.

[45] Struga M., Roszkowski P., Bielenica A., Otto-Ślusarczyk D., Stȩpień K., Stefańska J., Zabost A., Augustynowicz-Kopeć E., Koliński M., Kmiecik S. (2023). N-Acylated Ciprofloxacin Derivatives: Synthesis and In Vitro Biological Evaluation as Antibacterial and Anticancer Agents. ACS Omega.

[46] Azéma J., Guidetti B., Dewelle J., Le Calve B., Mijatovic T., Korolyov A., Vaysse J., Malet-Martino M., Martino R., Kiss R. (2009). 7-((4-Substituted)piperazin-1-yl) derivatives of ciprofloxacin: Synthesis and in vitro biological evaluation as potential antitumor agents. Bioorganic Med. Chem..

[47] Szostek T., Szulczyk D., Szymańska-majchrzak J., Koliński M., Kmiecik S., Otto-ślusarczyk D., Zawodnik A., Rajkowska E., Chaniewicz K., Struga M. (2022). Design and Synthesis of Menthol and Thymol Derived Ciprofloxacin: Influence of Structural Modifications on the Antibacterial Activity and Anticancer Properties. Int. J. Mol. Sci..

[48] Dileep K., Polepalli S., Jain N., Buddana S.K., Prakasham R.S., Murty M.S.R. (2018). Synthesis of novel tetrazole containing hybrid ciprofloxacin and pipemidic acid analogues and preliminary biological evaluation of their antibacterial and antiproliferative activity. Mol. Divers..

[49] Farooqi S.I., Arshad N., Perveen F., Channar P.A., Saeed A., Javed A. (2019). Aroylthiourea derivatives of ciprofloxacin drug as DNA binder: Theoretical, spectroscopic and electrochemical studies along with cytotoxicity assessment. Arch. Biochem. Biophys..

[50] Piplani M., Rajak H., Sharma P.C. (2017). Synthesis and characterization of N-Mannich based prodrugs of ciprofloxacin and norfloxacin: In vitro anthelmintic and cytotoxic evaluation. J. Adv. Res..

[51] Shahzad S.A., Sarfraz A., Yar M., Khan Z.A., Naqvi S.A.R., Naz S., Khan N.A., Farooq U., Batool R., Ali M. (2020). Synthesis, evaluation of thymidine phosphorylase and angiogenic inhibitory potential of ciprofloxacin analogues: Repositioning of ciprofloxacin from antibiotic to future anticancer drugs. Bioorganic Chem..

[52] Suresh N., Nagesh H.N., Sekhar K.V.G., Kumar A., Shirazi A.N., Parang K. (2013). Synthesis of novel ciprofloxacin analogues and evaluation of their anti-proliferative effect on human cancer cell lines. Bioorganic Med. Chem. Lett..

[53] Ali D.M.E., Aziz H.A., Bräse S., Al Bahir A., Alkhammash A., Abuo-Rahma G.E.-D.A., Elshamsy A.M., Hashem H., Abdelmagid W.M. (2024). Unveiling the Anticancer Potential of a New Ciprofloxacin-Chalcone Hybrid as an Inhibitor of Topoisomerases I & II and Apoptotic Inducer. Molecules.

[54] Kassab A.E., Gedawy E.M. (2018). Novel ciprofloxacin hybrids using biology oriented drug synthesis (BIODS) approach: Anticancer activity, effects on cell cycle profile, caspase-3 mediated apoptosis, topoisomerase II inhibition, and antibacterial activity. Eur. J. Med. Chem..

[55] Aziz H.A., El-Saghier A.M., Badr M., Elsadek B.E.M., Abuo-Rahma G.E.-D.A., Shoman M.E. (2024). Design, synthesis and mechanistic study of N-4-Piperazinyl Butyryl Thiazolidinedione derivatives of ciprofloxacin with Anticancer Activity via Topoisomerase I/II inhibition. Sci. Rep..

[56] Ahadi H., Emami S. (2020). Modification of 7-piperazinylquinolone antibacterials to promising anticancer lead compounds: Synthesis and in vitro studies. Eur. J. Med. Chem..

[57] Hardwidge P. (2017). Ciprofloxacin Conjugates as Potential Novel Antibiotics. https://www.semanticscholar.org/paper/Ciprofloxacin-Conjugates-as-Potential-Novel-Hardwidge/2e95930a8ed8aaf34e15ee4803062d2fbd4f16c1.

[58] Ahadi H., Shokrzadeh M., Hosseini-khah Z., Ghassemi Barghi N., Ghasemian M., Emadi E., Zargari M., Razzaghi-Asl N., Emami S. (2020). Synthesis and biological assessment of ciprofloxacin-derived 1,3,4-thiadiazoles as anticancer agents. Bioorganic Chem..

[59] Shi Z., Li Y., Kang Y., Hu G., Huang-fu C., Deng J.-B., Liu B. (2012). Piperonal ciprofloxacin hydrazone induces growth arrest and apoptosis of human hepatocarcinoma SMMC-7721 cells. Acta Pharmacol. Sin..

[60] Samir M., Ramadan M., Abdelrahman M.H., Abdelbaset M.S., Abourehab M.A.S., Abdel-Aziz M., Abuo-Rahma G.E.-D.A. (2021). 3,7-bis-benzylidene hydrazide ciprofloxacin derivatives as promising antiproliferative dual TOP I & TOP II isomerases inhibitors. Bioorganic Chem..

[61] Hu G.Q., Zhang Z.Q., Xie S.Q., Huang W.L. (2010). Synthesis and antitumor evaluation of C3/C3 fluoroquinolone dimers (I): Tethered with a fused heterocyclic s-triazolo [2,1-b][1,3,4]thiadiazole. Chin. Chem. Lett..

[62] Shaker S.A., Khaledi H., Cheah S.-C., Ali H.M. (2016). New Mn(II), Ni(II), Cd(II), Pb(II) complexes with 2-methylbenzimidazole and other ligands. Synthesis, spectroscopic characterization, crystal structure, magnetic susceptibility and biological activity studies. Arab. J. Chem..

[63] Adhikari S., Nath P., Das A., Datta A., Baildya N., Duttaroy A.K., Pathak S. (2024). A review on metal complexes and its anti-cancer activities: Recent updates from in vivo studies. Biomed. Pharmacother..

[64] Aldred K.J., Kerns R.J., Osheroff N. (2014). Mechanism of Quinolone Action and Resistance. Biochemistry.

[65] Sissi C., Palumbo M. (2009). Effects of magnesium and related divalent metal ions in topoisomerase structure and function. Nucleic Acids Res..

[66] Deweese J.E., Osheroff N. (2010). The Use of Divalent Metal Ions by Type II Topoisomerases. Metallomics.

[67] Uivarosi V. (2013). Metal Complexes of Quinolone Antibiotics and Their Applications: An Update. Molecules.

[68] Liu Y.-F., Ran S.-Y. (2020). Divalent metal ions and intermolecular interactions facilitate DNA network formation. Colloids Surf. B Biointerfaces.

[69] Patel M.N., Bhatt B.S., Dosi P.A., Amaravady N.V.R.L., Movaliya H.V. (2012). Synthesis, spectral investigation and biological interphase of drug-based cytotoxic square pyramidal coordination compounds. Appl. Organomet. Chem..

[70] Patel M.N., Patel C.R., Joshi H.N. (2013). Metal-based biologically active compounds: Synthesis, characterization, DNA interaction, antibacterial, cytotoxic and SOD mimic activities. Appl. Biochem. Biotechnol..

[71] Bykowska A., Starosta R., Jezierska J., Jeżowska-Bojczuk M. (2015). Coordination versatility of phosphine derivatives of fluoroquinolones. New CuI and CuII complexes and their interactions with DNA. RSC Adv..

[72] Hangas A., Aasumets K., Kekäläinen N.J., Paloheinä M., Pohjoismäki J.L., Gerhold J.M., Goffart S. (2018). Ciprofloxacin impairs mitochondrial DNA replication initiation through inhibition of Topoisomerase 2. Nucleic Acids Res..

[73] Ghilarov D.A., Shkundina I.S. (2012). DNA topoisomerases and their functions in a cell. Mol. Biol..

[74] Pommier Y., Sun Y., Huang S.N., Nitiss J.L. (2016). Roles of eukaryotic topoisomerases in transcription, replication and genomic stability. Nat. Rev. Mol. Cell. Biol..

[75] Papillon J., Ménétret J.-F., Batisse C., Hélye R., Schultz P., Potier N., Lamour V. (2013). Structural insight into negative DNA supercoiling by DNA gyrase, a bacterial type 2A DNA topoisomerase. Nucleic Acids Res..

[76] Aldred K.J., Schwanz H.A., Li G., McPherson S.A., Turnbough C.L., Kerns R.J., Osheroff N. (2013). Overcoming target-mediated quinolone resistance in topoisomerase IV by introducing metal-ion-independent drug-enzyme interactions. ACS Chem. Biol..

[77] Gupta D., Sachdeva E., Salman M., Kaur P., Gupta M.N., Kaur P., Sharma P. (2025). Chapter 9—Topoisomerases as targets for halting bacterial DNA replication. Bacterial Enzymes as Targets for Drug Discovery.

[78] Kokot M., Anderluh M., Hrast M., Minovski N. (2022). The Structural Features of Novel Bacterial Topoisomerase Inhibitors That Define Their Activity on Topoisomerase IV. J. Med. Chem..

[79] Emami S., Shafiee A., Foroumadi A. (2010). Quinolones: Recent Structural and Clinical Developments. Iran. J. Pharm. Res..

[80] Fief C.A., Hoang K.G., Phipps S.D., Wallace J.L., Deweese J.E. (2019). Examining the Impact of Antimicrobial Fluoroquinolones on Human DNA Topoisomerase IIα and IIβ. ACS Omega.

[81] Emami S., Shafiee A., Foroumadi A. (2006). Structural features of new quinolones and relationship to antibacterial activity against Gram-positive bacteria. Mini-Rev. Med. Chem..

[82] Mohamed M.F.A., Abuo-Rahma G.E.-D.A. (2020). Molecular targets and anticancer activity of quinoline–chalcone hybrids: Literature review. RSC Adv..

[83] Andreozzi G., Corvino A., Severino B., Magli E., Perissutti E., Frecentese F., Santagada V., Caliendo G., Fiorino F. (2024). Arylpiperazine Derivatives and Cancer: A New Challenge in Medicinal Chemistry. Pharmaceuticals.

[84] Asif M. (2015). Study of Antimicrobial Quinolones and Structure Activity Relationship of Anti-Tubercular Compounds. Res. Rev. J. Chem..

[85] Williams G.H., Stoeber K. (2012). The cell cycle and cancer. J. Pathol..

[86] Cross F.R., Buchler N.E., Skotheim J.M. (2011). Evolution of networks and sequences in eukaryotic cell cycle control. Philos. Trans. R. Soc. B Biol. Sci..

[87] Waseem A.M., Elmagzoub R.M., Abdelgadir M.M.M., Bahir A.A., EL-Gawaad N.S.A., Abdel-Samea A.S., Rao D.P., Kossenas K., Bräse S., Hashem H. (2024). An insight into the therapeutic impact of quinoxaline derivatives: Recent advances in biological activities (2020–2024). Results Chem..

[88] Aranha O., Wood D.P., Sarkar F.H. (2000). Ciprofloxacin mediated cell growth inhibition, S/G2-M cell cycle arrest, and apoptosis in a human transitional cell carcinoma of the bladder cell line. Clin. Cancer Res..

[89] Smart D.J., Halicka H.D., Traganos F., Darzynkiewicz Z., Williams G.M. (2008). Ciprofloxacin-induced G2 arrest and apoptosis in TK6 lymphoblastoid cells is not dependent on DNA double-strand break formation. Cancer Biol. Ther..

[90] Baum N., Schiene-Fischer C., Frost M., Schumann M., Sabapathy K., Ohlenschläger O., Grosse F., Schlott B. (2009). The prolyl cis/trans isomerase cyclophilin 18 interacts with the tumor suppressor p53 and modifies its functions in cell cycle regulation and apoptosis. Oncogene.

[91] Yadav V., Sultana S., Yadav J., Saini N. (2012). Gatifloxacin Induces S and G2-Phase Cell Cycle Arrest in Pancreatic Cancer Cells via p21/p27/p53. PLoS ONE.

[92] Wong R.S. (2011). Apoptosis in cancer: From pathogenesis to treatment. J. Exp. Clin. Cancer Res..

[93] Elmore S. (2007). Apoptosis: A review of programmed cell death. Toxicol. Pathol..

[94] Al-Wahaibi L.H., Elshamsy A.M., Ali T.F.S., Youssif B.G.M., Bräse S., Abdel-Aziz M., El-Koussi N.A. (2024). Design and Synthesis of New Dihydropyrimidine Derivatives with a Cytotoxic Effect as Dual EGFR/VEGFR-2 Inhibitors. ACS Omega.

[95] Khalil O.M., Gedawy E.M., El-Malah A.A., Adly M.E. (2019). Novel nalidixic acid derivatives targeting topoisomerase II enzyme; Design, synthesis, anticancer activity and effect on cell cycle profile. Bioorganic Chem..

[96] Mohamed A.H., Ahmed A.T., Al Abdulmonem W., Bokov D.O., Shafie A., Al-Hetty H.R.A.K., Hsu C.-Y., Alissa M., Nazir S., Jamali M.C. (2024). Interleukin-6 serves as a critical factor in various cancer progression and therapy. Med. Oncol..

[97] Alfaro C., Sanmamed M.F., Rodríguez-Ruiz M.E., Teijeira Á., Oñate C., González Á., Ponz M., Schalper K.A., Pérez-Gracia J.L., Melero I. (2017). Interleukin-8 in cancer pathogenesis, treatment and follow-up. Cancer Treat. Rev..

[98] Tatsuno R., Ichikawa J., Komohara Y., Pan C., Kawasaki T., Enomoto A., Aoki K., Hayakawa K., Iwata S., Jubashi T. (2024). Pivotal role of IL-8 derived from the interaction between osteosarcoma and tumor-associated macrophages in osteosarcoma growth and metastasis via the FAK pathway. Cell Death Dis..

[99] Hirano T. (2021). IL-6 in inflammation, autoimmunity and cancer. Int. Immunol..

[100] Grebenciucova E., VanHaerents S. (2023). Interleukin 6: At the interface of human health and disease. Front. Immunol..

[101] Soler M.F., Abaurrea A., Azcoaga P., Araujo A.M., Caffarel M.M. (2023). New perspectives in cancer immunotherapy: Targeting IL-6 cytokine family. J. Immunother. Cancer.

[102] Yang H., Karl M.N., Wang W., Starich B., Tan H., Kiemen A., Pucsek A.B., Kuo Y.-H., Russo G.C., Pan T. (2022). Engineered bispecific antibodies targeting the interleukin-6 and -8 receptors potently inhibit cancer cell migration and tumor metastasis. Mol. Ther..

[103] Hashem H., Hassan A., Abdelmagid W.M., Habib A.G.K., Abdel-Aal M.A.A., Elshamsy A.M., El Zawily A., Radwan I.T., Bräse S., Abdel-Samea A.S. (2024). Synthesis of New Thiazole-Privileged Chalcones as Tubulin Polymerization Inhibitors with Potential Anticancer Activities. Pharmaceuticals.

[104] Gudimchuk N.B., McIntosh J.R. (2021). Regulation of microtubule dynamics, mechanics and function through the growing tip. Nat. Rev. Mol. Cell Biol..

[105] Wang X., Gigant B., Zheng X., Chen Q. (2023). Microtubule-targeting agents for cancer treatment: Seven binding sites and three strategies. MedComm–Oncol..

[106] Wordeman L., Vicente J.J. (2021). Microtubule Targeting Agents in Disease: Classic Drugs, Novel Roles. Cancers.

[107] Zhang Q., Qin Y., Zhao J., Tang Y., Hu X., Zhong W., Li M., Zong S., Li M., Tao H. (2019). Thymidine phosphorylase promotes malignant progression in hepatocellular carcinoma through pentose Warburg effect. Cell Death Dis..

[108] Bijnsdorp I.V., Capriotti F., Kruyt F.A.E., Losekoot N., Fukushima M., Griffioen A.W., Thijssen V.L., Peters G.J. (2011). Thymidine phosphorylase in cancer cells stimulates human endothelial cell migration and invasion by the secretion of angiogenic factors. Br. J. Cancer.

[109] Warfield B.M., Reigan P. (2022). Multifunctional role of thymidine phosphorylase in cancer. Trends Cancer.

[110] Teleanu R.I., Chircov C., Grumezescu A.M., Teleanu D.M. (2019). Tumor Angiogenesis and Anti-Angiogenic Strategies for Cancer Treatment. J. Clin. Med..

[111] Lopes-Coelho F., Martins F., Pereira S.A., Serpa J. (2021). Anti-Angiogenic Therapy: Current Challenges and Future Perspectives. Int. J. Mol. Sci..

[112] Emran T.B., Shahriar A., Mahmud A.R., Rahman T., Abir M.H., Siddiquee M.F.-R., Ahmed H., Rahman N., Nainu F., Wahyudin E. (2022). Multidrug Resistance in Cancer: Understanding Molecular Mechanisms, Immunoprevention and Therapeutic Approaches. Front. Oncol..

[113] Duan C., Yu M., Xu J., Li B.-Y., Zhao Y., Kankala R.K. (2023). Overcoming Cancer Multi-drug Resistance (MDR): Reasons, mechanisms, nanotherapeutic solutions, and challenges. Biomed. Pharmacother..

[114] Kurimchak A.M., Herrera-Montávez C., Montserrat S., Araiza D., Hu J., Jin J., Duncan J.S. (2021). MDR1 Drug Efflux Pump Promotes Intrinsic and Acquired Resistance to PROTACs in Cancer Cells. bioRxiv.

[115] Al-Lazikani B., Banerji U., Workman P. (2012). Combinatorial drug therapy for cancer in the post-genomic era. Nat. Biotechnol..

